# Multimodal imaging in choroidal osteoma

**DOI:** 10.1186/s40942-018-0132-0

**Published:** 2018-08-15

**Authors:** Francisco Olguin-Manríquez, Ana Bety Enríquez, Nicolás Crim, Miroslava Meraz-Gutierrez, Vidal Soberón-Ventura, Ismael Ávila, Virgilio Morales-Canton, Juan Manuel Jimenez-Sierra

**Affiliations:** 1 “Dr. Luis Sánchez Bulnes” Hospital from Asociación Para Evitar la Ceguera en México I.A.P, Vicente García Torres 46, Colonia Barrio de San Lucas, Delegación Coyoacón, 04030 Mexico City, Mexico; 2 Retina Department, “Dr. Luis Sánchez Bulnes” Hospital from Asociación Para Evitar la Ceguera en México I.A.P, Mexico City, Mexico

**Keywords:** Choroidal osteoma, Choroidal excavation, Choroidal neovascularization, EDI-OCT, OCT-A, Ultra-widefield retinal imaging

## Abstract

**Purpose:**

To describe characteristics of choroidal osteomas (CO), using ocular ultrasound, fluorescein angiography, ultra-widefield retinal imaging, ultra-widefield autofluorescence, optical coherence tomography, enhanced-depth-imaging OCT, and OCT angiography (OCT-A).

**Methods:**

Retrospective, observational case series study. Clinical records from patients with diagnosis of CO who underwent complete imaging evaluation were analyzed.

**Results:**

Sixteen eyes from 11 patients were included. Mean patient age was 33.4 years (range 20–61), 72.7% were female, 100% were Hispanic, and 54.5% had unilateral CO. Median visual acuity was 20/150 (range 20/20–2000). CO was completely calcified in 25%, partially decalcified in 50%, and decalcified in 25%. Other features included choroidal neovascularization (18.75%), focal choroidal excavation (12.5%), choroidal depression associated to decalcification (18.75%), thinning of outer retina and photoreceptor layers over decalcified tumor (75%). Decreased fluorescence on FAF was observed in decalcified regions while relatively preserved fluorescence was observed in calcified regions.

**Conclusions:**

Nowadays, diagnostic tests provide important information about each stage of choroidal osteoma. Progressive decalcification of the tumor might have a common pathogenic role for development of FCE or choroidal depression. OCT-A/FA proved to be valuable tools for detection of CNV in patients with CO.

## Introduction

Choroidal osteoma (CO) is a rare benign tumor of the choroid, which is composed of mature bone (trabecular and/or compact) and vascular channels [1–4]. Gass et al. made the first description of this kind of neoplasm in 1978, and since then multiple case reports and series have been published [5].

CO is typically unilateral (80% of cases) and it usually affects young healthy female patients [6]. It arises in late childhood or early adulthood and its most common symptoms are blurred vision, metamorphopsia and presence of a scotoma [7]. The clinical appearance of the tumor may vary from white-cream or yellow-gray to orange, well-defined, which according to some authors corresponds to the grade of calcification (orange pigmentation is present in areas with more ossification) [6].

Over time, ocular ultrasound (US), fluorescein angiography (FA) and optical coherence tomography (OCT) have been widely used for diagnosis and follow-up of CO. Enhanced depth imaging OCT (EDI-OCT) is a recent addition of OCT, that has been able to reveal the presence of bone lamella, tubular lamella with optically empty center, vascular channels and trabecular bone in patients with CO [8, 9].

In patients with CO, OCT angiography (OCT-A) is a new non-invasive imaging technique that employs motion contrast from blood flow to generate high-resolution angiographic images, in patients with CO has been able to show a dense irregular vascular network in the outer retinal layer (ORL) and choroid capillary layers [10]. On the other hand OCT-A [11].

The aim of this study is to describe the morphology of CO using a multimodal image system.

## Methods

The clinical records of patients with a diagnosis of choroidal osteoma who had undergone multimodal fundus imaging on the retina service at Asociación para Evitar la Ceguera en México were reviewed. The diagnosis was based on the presence of a yellow-white to orange-red mass deep to the RPE and bone density on ultrasonography. Institutional review board approval was obtained for this retrospective study.

Patient data were extracted from medical records and included age at diagnosis, gender (male, female), chief complaint, ocular comorbidities. Ophthalmic features included best-corrected visual acuity (BCVA), tumor laterality (unilateral or bilateral), location (foveal, extrafoveal).

Multimodal imaging analysis included ultrasonography (US), fluorescein angiography (FA), ultra-widefield retinal imaging (UWF), ultra-widefield autofluorescence (UWF-FAF), optical coherence tomography (OCT) and OCT angiography (OCT-A).

Enhanced depth imaging optical coherence tomography (Spectralis HRA + OCT; Heidelberg Engineering, Germany), data included tumor surface configuration (flat or depressed), effects of tumor on overlying retina (RPE, photoreceptor and inner retina status). One independent physician manually measured osteoma thickness with a caliper function through the epicenter of the tumor.

Ultra-wide field color fundus photograph and ultra-widefield fundus autofluorescence (Optos Daytona; Optos PLC, United Kingdom) data included tumor location (foveal, extrafoveal), tumor color (yellow, orange, white), and fundus autofluorescence pattern. Decalcification Calcification (complete, partial) was defined as pale areas within the osteoma, RPE thinning and visibility of underlying choroidal vessels.

Fluorescein angiography (FA) (Spectralis HRA + OCT; Heidelberg Engineering, Germany) data included presence or absence of CNV. OCT-A images were analyzed in patients in whom FA was performed and correlated with the presence or absence of CNV (SS OCT Angio; Topcon Corporation, Japan).

## Results

There were 16 eyes in 11 patients with choroidal osteoma included in this study. The demographic and clinical characteristics are summarized in Table 1. All patients were Hispanic and diagnosis corresponded to primary CO in 15 eyes, whereas one patient had CO secondary to choroidal hemangioma. The median age at presentation was 33.4 years (range 20–61 years). Most patients were female (72.7%).

**Table 1:** Clinical and imaging characteristics of choroidal osteomas

Case	Age	Gender	Laterality	Localization		Decalcification		BCVA (Snellen)		Opthalmic conditions	Clinically suspected CNV		Autofluorence		FA		OCT-A findings (superficial choroid)	
				RE	LE	RE	LE	RE	LE		RE	LE	RE	LE	RE	LE	RE	LE
1	21	F	L	DNA	XF	WNL	C	20/150	20/200	Keratoconus	N	N	WNL	H-hd	ND	ND	WNL	AV
2	20	M	B	SF	SF	PC	PC	20/800	20/200	None	Y	Y	h-He	h-Hd	WD	WD	DB	DB
3	21	F	B	SF	SF	C	PC	20/20	20/20	None	Y	Y	H-hd	H-he	CNV	WD	CNV	VN
4	27	F	R	XF	DNA	C	WNL	20/20	20/50	Keratoconus	N	N	WNL	ND	ND	ND	ND	ND
5	30	F	R	SF	DNA	PC	WNL	20/50	20/25	None	N	N	h-Hd	WNL	WD	WNL	VN	WNL
6	43	M	B	SF	SF	DC	DC	20/150	20/200	None	N	N	H-hd	H-hd	ND	ND	ND	ND
7	61	F	L	DNA	XF	WNL	PC	20/20	20/25	None	N	N	WNL	H-hd	WNL	WD	WNL	VN
8	42	F	B	SF	XF	PC	PC	20/200	20/20	None	Y	Y	h-Hd + h-He	h-He	CNV	CNV	CNV	CNV
9	38	M	L	DNA	XF	WNL	C	20/20	20/30	CSC	N	Y	WNL	h-He	WNL	CSC	WNL	VN
10	30	F	B	SF	SF	DC	DC	20/2000	20/2000	None	N	N	h-He	h-He	WD	WD	ND	ND
11	35	F	L	DNA	SF	WNL	PC	20/20	20/150	None	N	N	WNL	h-Hd	WNL	WD	ND	ND

*F* female, M male, *L* left, *R* right, *B* bilateral, *RE* right eye, *LE* left eye, *NDNA* does not apply, *SF* subfoval, *XF* extrafoveal, *WNL* whithin normal limits, *C* calcified, *PC* partially decalcified, *DC* decalcified, *BCVA* best corrected visual acuity, *CSC* central serous chorioretinopathy, *CNV* choroidal neovascularization, *N* no, *Y* yes, *h-He* hypo-autofluorescent with hyper-autofluorescent edge, *H-hd* hyper-autofluorescent with hypo-autofluorescent dots, *h-Hd* hypo-autofluorescent with hyper-autofluorescent dots, *H-he* hyper-autofluorescent with hypo-autofluorescent edge, *FA* fluorescein angiography, *ND* not done, *WD* window defect, OCT-A Optical coherence tomography angiography, *DB* dark background where decalcification was present, *VN* vascular network within tumor, *AV* absence of vascular flow within tumor

Initial symptoms included blurred vision [9 patients, (82%)], metamorphopsia [1 patient, (9%)], asymptomatic [1 patient, (9%)]. Ocular conditions that accompanied the diagnosis of CO were keratoconus (2 patients) and central serous chorioretinopathy (1 patient). Visual acuity was 20/20–20/50 in 7 eyes (44%), 20/60–20/150 in 2 eyes (12%), 20/200 or worse in 7 eyes (44%). Poor visual acuity (20/200 or worse) was related to foveal photoreceptor loss overlying deossified osteoma (n = 6), subfoveal choroid neovascular membrane (n = 1), keratoconus (n = 1).

Five patients (45.5%) had bilateral CO; while the other 54.5% had unilateral CO. The osteoma was completely calcified in 4 eyes (25%), partially decalcified in 8 eyes (50%) and decalcified in 4 eyes (25%). Tumor location was subfoveal in 12 eyes (75%); extrafoveal in 4 eyes (25%).

CO showed different FAF patterns, which we classified as normal autofluorescence (isoautofluorescent, 6.25%), predominantly hyper-AF (37.5%) and predominantly hypo-AF (56.25%); decreased fluorescence on FAF was observed in decalcified tumoral regions while relatively preserved fluorescence was observed in calcified regions. Patients with worse visual acuity (≤ 20/200) presented predominantly hypo-AF pattern (5 eyes, 31.25%).

OCT data demonstrated a mean central foveal thickness of 265.5 μm (range 101–599 μm), a mean subfoveal choroidal thickness of 498.17 μm (range 288–736), and a mean central tumor thickness of 574.86 μm (range 246–1084). Patients with decalcified portion of tumor displayed and overlying thinned inner retinal layers in 4 eyes (25%), thinned outer retina with thinned to absent photoreceptor layer in 12 eyes (75%), and overlying RPE hyperplasia in 3 eyes (25%).

Seven eyes with clinical suspicion of choroidal CNV were imaged with FA and OCT-A. Leakage of fluorescein dye was present in 2 patients (3 eyes, 18.75%); OCT-A made evident the location of abnormal vascular network in outer retina and choriocapillaris segmentation. The presence of CNV was excluded in 4 patients using FA & OCT-A.

OCT-A analysis showed the following 4 patterns: absence of vascular flow within tumor (6.25%), dark background where decalcification was present (12.5%), vascular network within tumor (25%), and presence of a neovascular membrane (18.75%).

## Discussion

**Fig. 1:**
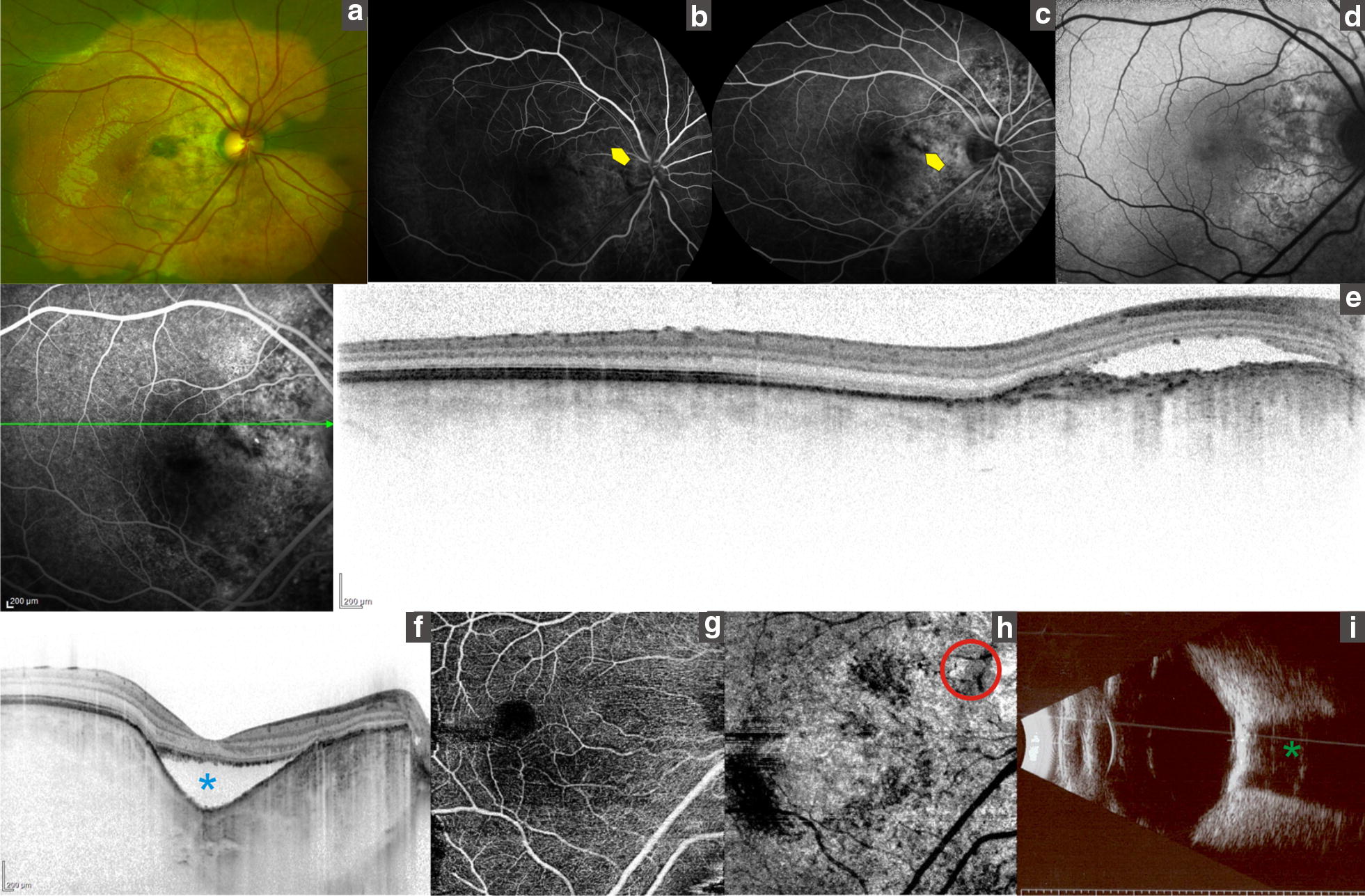
Multimodal imaging in choroidal osteoma. **a** Fundus photograph shows a flat well-demarcated orange lesion in the macular area. **b**, **c**. Fluorescein angiogram showing early hyperfluorescence and quiescent late staining in the yuxtapapillary area (yellow arrow). **d** FAF showing predominantly hyper-AF in the juxtapapillary area. **e**, **f**. OCT-EDI showing a focal choroidal excavation (asterisk). **g**, **h** OCT-A Boundaries of quiescent CNV in deep plexus (red circle). **i** B-scan ultrasonography consistent with CO (green asterisk)

**Fig. 2:**
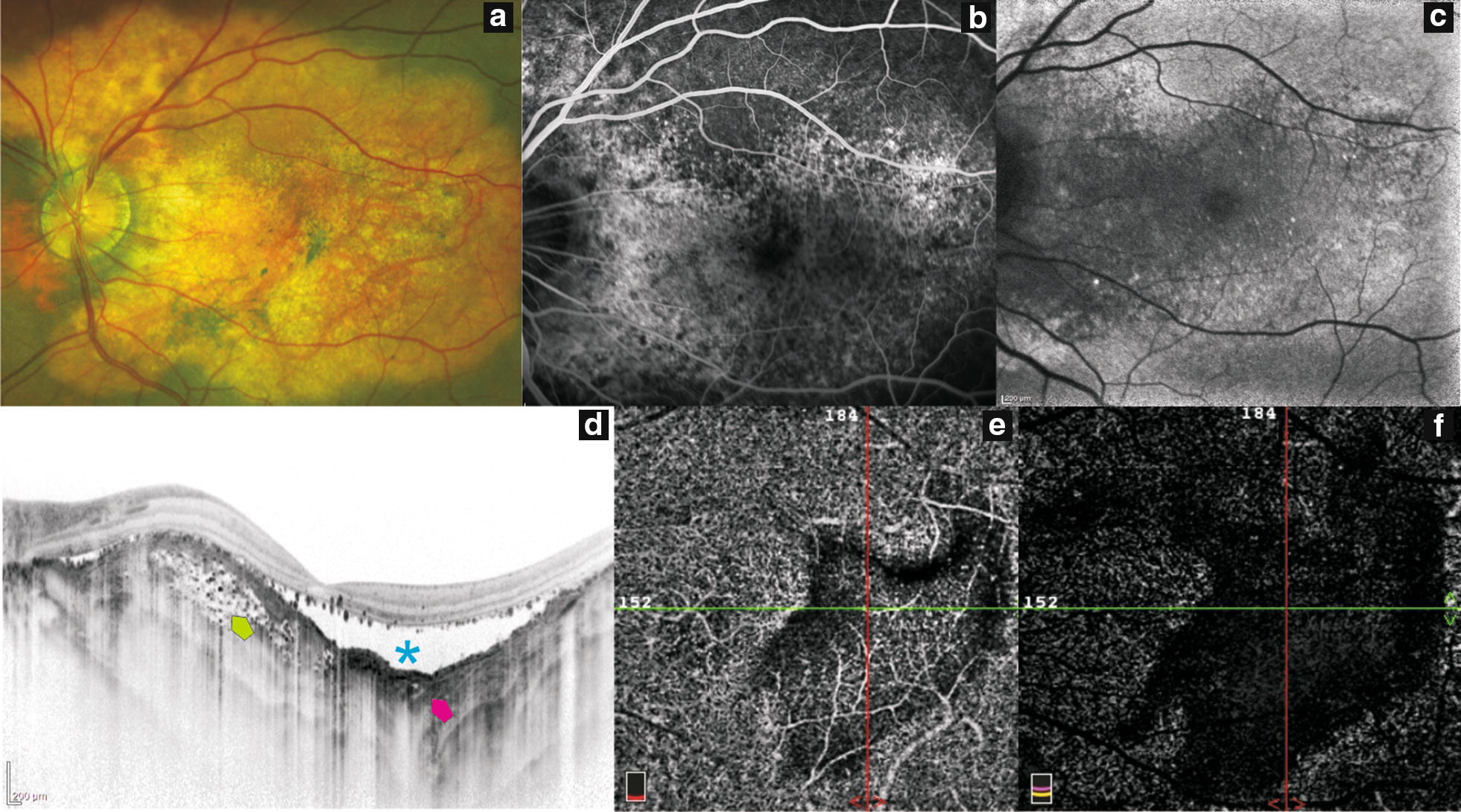
Multimodal imaging in choroidal osteoma. **a** Fundus photograph showing an orange-yellow plaque in the macular region. **b** Fluorescein angiography showing hyperfluorescence due to damage of the retinal pigment epithelium over a partially ossified tumor (yellow plaque). **c** Blue-light autofluorescence (bAF) showing predominantly hypo-AF with hyper-AF dots within the tumor. **d** OCT EDI showing a concave formation within decalcified portion of tumor (asterisk); calcified tumoral regions have multiple hyperreflective dots surrounding hyporeflective spaces (green arrow). **e**, **f**. OCT-A. Superficial and deep vascular network showing dark background were the decalcification is present

**Fig. 3:**
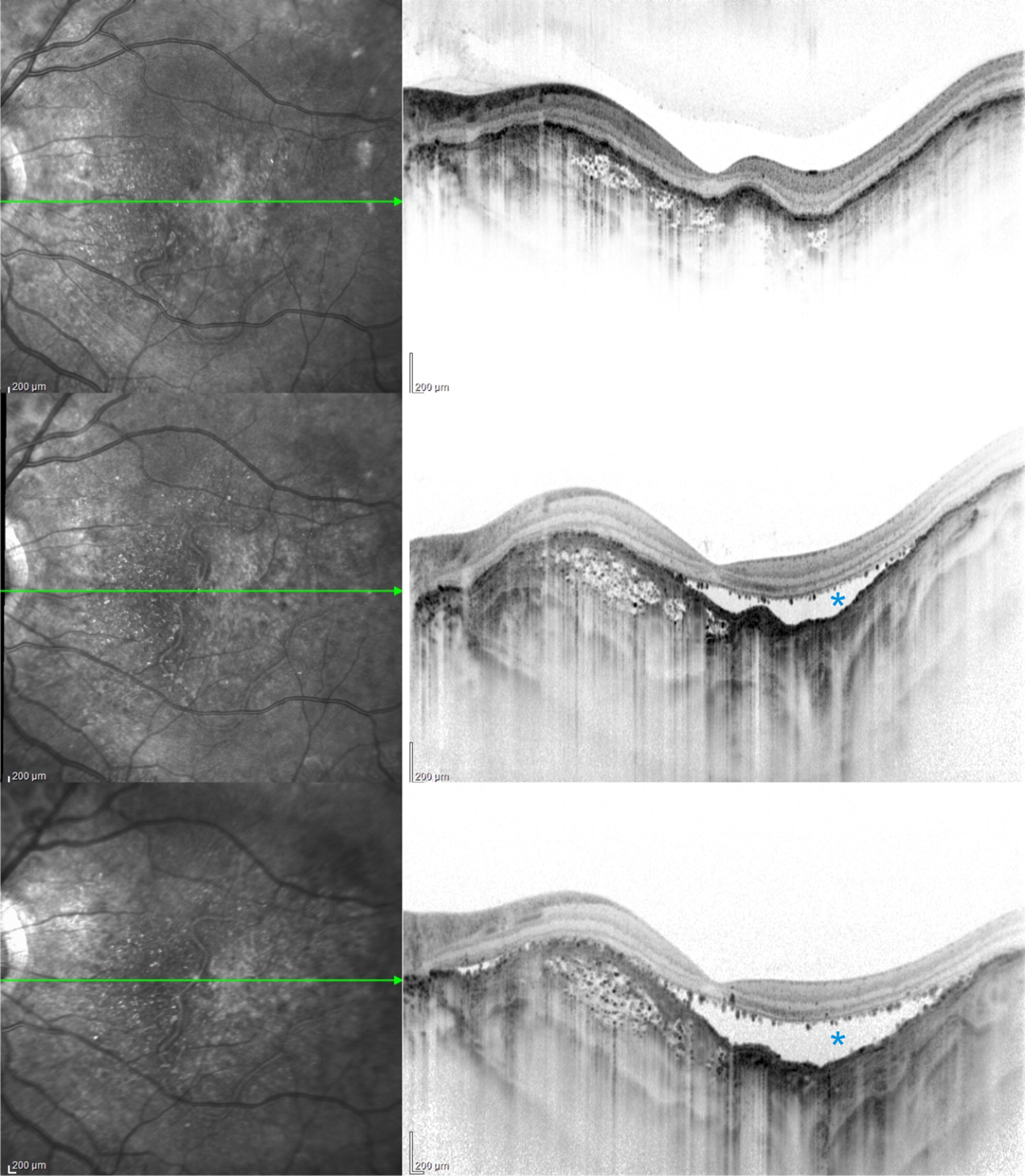
Choroidal depression associated with tumor decalcification. Top: Optical coherence tomography progression of a patient with partially calcified CO. Middle and bottom: Choroidal vessels become prominent in decalcified areas. RPE-photoreceptor detachment induced by decalcification (asterisk). These. Images were taken after 13 and 17 months, respectively

Choroidal osteomas may demonstrate decalcification, CNV, retinal pigment epithelium (RPE) alterations and vision loss [6]. Patients with calcified areas, even subfoveolar ones, had better visual acuities; while patients with decalcified CO had lower visual acuities correlated with RPE disruption and outer layer thinning and photoreceptor loss, and corresponded to hypo-AF on AF [8, 12]. Table 1. In our series two eyes (12.5%) presented CNV in the proximity of focal choroidal excavation (FCE) Fig. 1. Margolis et al. [13] described the FCE in conforming lesions, in which the overlying retina is close to the RPE, and nonconforming lesions in which a hypo reflective space is visible between the retina and RPE, in our series one patient had conforming and the other one had nonconforming FCE. Pierro et al. [14] described in two patients with CO the presence of CNV and FCE. CE has an increased separation between retinal pigment epithelium and neurosensory retina without schisis of the corresponding retinal layers; the location is in correspondence or in proximity of the tumor. In our series three eyes had choroidal depression associated with tumor decalcification Fig. 2. This feature is characterized by intrinsic hyperreflective dots within tumor (speckled regions) in the proximity of choroidal depression, neurosensory retina may show schisis, this depression is not always associated with CNV. FCE and choroidal depression may represent distinct stages of focal decalcification of the tumor, this hypothesis is supported by the fact that we were able to follow one patient that developed choroidal concavity, situation that allowed us to see that this depression can grow overtime (Fig. 3).

## Conclusions

Choroidal osteoma is an ossifying tumor involving the choroid, its natural course may include tumor growth, calcification and decalcification; visual acuity depends on choroidal neovascularization and retinal changes associated to decalcification [12]. Duration of this condition is a mayor risk factor associated with vision loss. After 10 years, approximately 51% manifest evidence of growth and nearly 50% showed decalcification. Calcified and decalcified areas have demonstrated changes in outer retina. Optical coherence tomography changes have been shown that calcified areas have intact outer retina whereas decalcified portion have thinned to absent outer retina and photoreceptor layers [12].

In our series, the mean age at diagnosis was 33 years and females represented 72.7%, however 45.5% of our patients were bilateral, this patients showed osteomas located in the macular area with extension beyond the vascular arcades and showed RPE alterations due to osteoma decalcification.

Patients witth CNV whose medical records had FA and OCT-A also where evaluated, both have good correlation in determining the site of neovascularization.

Choroidal excavations observed in this series correlate with previous descriptions made by Jampol et al. and Wakabayashi et al. [15, 16]. Table 2. To our best knowledge Pierro et al. [14] described two patients with CO and FCE in the proximity of CNV. In this series two eyes had CNV in the proximity of FCE and one eye had FCE in the boundaries of the osteoma and normal choroidal tissue.

**Table 2:** Literature review of case reports analyzing choroidal osteoma characteristics

Authors	Year	n (eyes)	Results
Shields et al. [12]	2007	22	OCT: calcified portion displayed an intact inner retina, outer retina and photoreceptors, but decalcified portion showed intact inner retina with thinned or absent outer retina and photoreceptors. BCVA was better in eyes with calcified osteomas
Margolis et al. [13]	2011	13	AF: hypoautofluorescence. Indocyanine green angiography showed relative hypofluorescence. SD-OCT: separation between outer retina and RPE within the excavation; and other cases in which the outer retina layers conform to the retina pigment eccccccpithelium within the excavation. Choroidal thickness of uninvolved choroid was thicker than normal
Freton et al. [9]	2011	11	SD-OCT: different reflectivity pattern among hyporeflective, isoreflective and hyperreflective, besides retina exhibited degenerative changes
Navajas et al.	2012	3	FD-OCT show in calcified tumors a distinctive latticework pattern resembling a spongy bone structure, decalcified areas show hyperreflective areas above Bruch membrane and absence of choroidal vessels. AF: Decalcified tumor had reduced over all fluorescence
Shields et al. [11]	2015	15	EDI-OCT: horizontal lamellar lines, hyperreflective horizontal lines, horizontal and vertical tubular lamella. Photoreceptors were intact in ossified tumors meanwhile those were atrophic or thinning in deossified osteomas
Pierro et al. [14]	2017	3	FCE and CNV in CO. OCT-A is a useful skill to detect CNV
Cennamo et al. [17]	2017	6	OCT-A: fine vascular network within the tumor. EDI-OCT: horizontal lamellar lines, horizontal and vertical tubules and speckled regions. B-Scan echography: solid mass with acoustic shadowing

*FCE* focal choroidal excavation, *CNV* choroidal new vascularization, *OCT-A* angiography optical coherence tomography, *FD-OCT* Fourier domain optical coherence tomography, *AF* autofluorescence, *SD-OCT* spectral domain optical coherence tomography, *RPE* retinal pigment epithelium, *EDI-OCT* enhance depth imaging optical coherence tomography, *BCVA* best corrected visual acuity

Although several authors have already described clinical and OCT characteristics of CO, to our best knowledge this is the first case series report of multimodal imaging findings of CO in Hispanic patients. Because this is a rare pathology, the number of cases reported in the literature is scarce, and this also constituted a limitation to our study. However, we believe these findings can deepen the information about the behavior of this uncommon tumor.

## Abbreviations

CO: choroidal osteoma
CNV: choroidal neovascularization
OCT: optical coherence tomography
EDI-OCT: enhanced depth imaging optical coherence tomography
OCT-A: optical coherence tomography angiography
UWF: ultra-widefield retinal imaging
UWF-FAF: ultra-widefield fundus autofluorescence
FA: fluorescein angiography
FCE: focal choroidal excavation
APEC: Hospital “Dr. Luis Sánchez Bulnes” from the Asociación para Evitar la Ceguera en México I.A.P
BCVA: best-corrected visual acuity
